# The unregulated majority: who ensures quality in non-submission real-world studies?

**DOI:** 10.3389/fmed.2026.1771760

**Published:** 2026-03-02

**Authors:** Alexandros Sagkriotis

**Affiliations:** 1 Independent Consultant, Real-World Evidence and Health Data Science, London, United Kingdom; 2 Helios Academy Limited, London, United Kingdom

**Keywords:** analytical transparency, governance, non-submission studies, observational research, pharmaceutical industry, protocol registration, real-world evidence (RWE), scientific integrity

## Abstract

Real-world evidence (RWE) plays an increasingly important role in healthcare decision-making, yet most industry-sponsored RWE studies are not intended for regulatory or reimbursement submission. While many such studies are conducted rigorously, this non-submission evidence base often operates without consistent protocol pre-specification, analytical transparency, or structured oversight, creating variability in quality and credibility. This Policy Brief examines this governance blind spot and argues that the challenge is structural rather than individual or intentional. Drawing on regulatory pilots and harmonisation initiatives, it shows that tools to strengthen non-submission RWE already exist but are unevenly applied. A framework is proposed based on three pillars: protocol registration, analytical transparency, and proportionate internal governance, including accountability for patient-contributed data. Extending these safeguards beyond submission-grade studies would raise baseline RWE quality and strengthen trust in RWE.

## Introduction

Real-world evidence (RWE) has become an increasingly prominent element of modern healthcare decision-making, moving from a marginal complement to randomized controlled trials (RCTs) into a central pillar of regulatory, reimbursement, and clinical deliberations. The European Medicines Agency (EMA), the United States Food and Drug Administration (FDA), and the United Kingdom’s Medicines and Healthcare products Regulatory Agency (MHRA) have all released frameworks to encourage the use of real-world data (RWD) under conditions of relevance, reliability, and transparency (1, 2). In this context, it is important to distinguish among different real-world data sources. Disease registries, electronic medical records (EMRs), and administrative claims vary substantially in completeness, governance, and analytical flexibility. This commentary focuses on the quality and governance of industry-sponsored, non-submission RWE that uses these data for communication, medical education, or exploratory purposes. Recent initiatives such as the EMA pilot on regulatory use cases (3) and multi-country lifecycle evidence generation programs (4) demonstrate that when RWE is generated under regulatory oversight, it can meaningfully inform safety surveillance, paediatric and rare disease research, and trial feasibility. Complementing these regulatory initiatives, the EMA’s *Clinical Evidence 2030* perspective envisions a system where patient voice, transparency, and advanced analytics drive evidence generation across the lifecycle (5).

At the same time, methodological innovation has expanded the credibility of observational research. Target trial emulation approaches (6), harmonization pilots in oncology (7, 8), and innovative external comparator strategies such as blinded expert video review in rare diseases (9) illustrate how RWE can approach trial-level rigor. Parallel efforts to improve reporting and transparency—such as the STaRT-RWE and HARPER templates, the REQueST tool, and the EU push toward registry qualification—further show that tools to strengthen trust in RWE are already available (10, 11). International harmonisation initiatives, including those led by Duke Margolis and the International Council for Harmonisation (ICH), have sought to align definitions of reliability, relevance, and quality across agencies (12). Industry stakeholders, through EFPIA and other consortia, have also called for clear good practice principles in the conduct of non-interventional studies (NIS), including mandatory registration, bias reduction strategies, and adherence to FAIR data standards (13).

This paper does not argue that non-submission real-world evidence is inherently low quality, nor that industry actors lack scientific intent. Many non-submission studies are conducted rigorously and have generated valuable insights into disease burden, treatment patterns, and unmet medical need. Rather, this commentary identifies a structural governance gap, driven by incentives, timelines, and uneven oversight, in which the absence of consistent safeguards allows wide variability in study quality. The challenge addressed here is therefore systemic rather than individual, and concerns how baseline standards of transparency, accountability, and scientific integrity can be applied proportionately across the full real-world evidence ecosystem.

Despite these advances, however, a paradox remains. The majority of RWE produced in the pharmaceutical sector is not designed for submission to regulatory or health technology assessment (HTA) authorities. Instead, it is generated for scientific communication, burden-of-disease description, medical education, or indirect support of product positioning (13). While similar quality concerns can affect RWE generated by academia, payers, or not-for-profit entities, this commentary focuses on pharmaceutical-sponsored work because of its scale, visibility, and influence on clinical perception. Such studies—which dominate conference abstracts, poster presentations, and local registry analyses—frequently lack prespecified protocols, comparator groups, or peer-reviewed oversight. For clarity, the term “non-submission RWE” is used throughout this paper to describe studies not originally designed or prospectively planned for formal regulatory or HTA submission. In practice, some such studies may later inform reimbursement dialogue, economic model assumptions, or policy discussions. The distinction is therefore functional rather than absolute, and the quality principles discussed here apply equally to evidence that ultimately enters regulatory or reimbursement contexts.

Recent reviews confirm that even in leading journals, over a quarter of observational studies fail to report key quality elements such as missing-data handling, residual confounding, or falsification tests (14). Audits in dermatology and other therapeutic areas have demonstrated persistent weaknesses in reporting sample size justification, attrition, and data completeness, despite the availability of STROBE guidelines (15). From a reviewer’s standpoint, weaknesses in question specification, study design, and data fitness remain common, even when target trial reconstruction is attempted (16).

Evidence from comparative analyses of RWE versus RCTs reinforces the scale of the challenge. For example, work comparing diabetic kidney disease trial populations with real-world cohorts revealed minimal overlap, highlighting how unadjusted comparisons can be misleading when data generation mechanisms differ fundamentally (17). In oncology, broadening trial eligibility criteria and integrating real-world populations has been championed to enhance representativeness and external validity (18). Similarly, proposals for adaptive and biomarker-driven designs, pragmatic registries, and digital endpoints underscore how high-quality RWE can complement trials in precision medicine (19). Extending these principles to purely RWD-based studies requires emulating comparable methodological rigor—pre-specifying design elements, analytic populations, and endpoints—even when no randomized trial exists. The same logic of target-trial emulation and bias minimization can strengthen registry-, EMR-, or claims-based RWE that stands alone. Yet these methodological insights remain largely confined to high-stakes regulatory or academic settings. In the broader landscape of non-submission industry-sponsored RWE, study quality is often compromised by speed, feasibility considerations, or promotional intent.

The contrast is stark: while regulators and academic consortia invest heavily in improving the credibility of RWE, the vast majority of studies produced for communication, education, or awareness operate with little to no structured oversight. This “unregulated majority” presents not only a scientific shortfall but also an ethical challenge. Patients contribute data—directly or indirectly—without assurance that the resulting evidence meets minimal standards of validity or transparency. In practice, patients contribute either directly through registry participation and patient-reported outcomes or indirectly via electronic health records, insurance claims, and linked digital health sources. The time has come to extend governance and quality safeguards beyond the narrow scope of regulatory submissions, ensuring that all RWE intended to influence clinical understanding or decision-making meets basic principles of scientific integrity.

## The blind spot: recurring quality issues in non-submission RWE

The dominance of non-submission RWE within the pharmaceutical ecosystem is both striking and underappreciated. These studies include disease burden descriptions, treatment pattern analyses, pragmatic registry reports, and exploratory effectiveness comparisons that rarely proceed to formal submission. Yet they form the majority of the evidence landscape encountered by clinicians at congresses, in medical affairs slide decks, and in peer-reviewed but low-impact journals (typically those with an impact factor <2 or not indexed in major bibliographic databases) (13, 14). Their prevalence makes their weaknesses all the more concerning.

Several recurring flaws characterize this body of work:

1. *Protocol shortcuts*: Many non-submission studies advance with little more than a concept slide or one-page outline rather than a detailed, prespecified protocol. In reviews of congress abstracts, up to one-third of non-submission studies are based only on concept notes or short outlines rather than full protocols. Without proper adjustment, comparators, or sensitivity analyses. This absence of planning limits reproducibility and increases the risk of selective analytic choices (1, 13).
2. *Descriptive overreach*: Studies intended to be descriptive—such as treatment pattern reviews—are often stretched to make causal inferences about effectiveness or safety without proper adjustment, comparators, or sensitivity analyses. Even when causal inferences are attempted, confidence intervals and sensitivity analyses are rarely presented to contextualize findings. In oncology, proxy endpoints like time-to-next-treatment (TTNT) are commonly used in place of validated outcomes, yet rarely accompanied by discussion of their limitations (16).
3. *Selective reporting*: Non-submission RWE is disproportionately channelled into congress abstracts and posters. Positive findings are highlighted, while neutral or negative analyses are quietly omitted. As an example, the dermatology field offers repeated examples of registry data being presented selectively, emphasizing supportive comorbidity profiles without rigorous confounder adjustment (15).
4. *Methodological issues*: Other recurrent flaws include poor data quality and governance, unaddressed selection bias, intercurrent confounding, and immortal time bias—all of which compromise reliability if not explicitly managed.
5. *Weak peer review*: Weak peer review is a common problem, since the majority of these outputs are confined to conference proceedings where peer review is minimal, if it occurs at all, while many observational studies published in low-threshold journals also fail to meet transparency and reproducibility standards (13, 14). By weak peer review we refer to acceptance processes without statistical or methodological scrutiny, typical of many conference proceedings and lower-tier journals.
6. *Promotional drift:* In some instances, studies may be designed with dual scientific and strategic objectives, which can create tension between neutral inquiry and product positioning. Registries created in parallel with product launches often highlight unmet needs or under-treatment patterns that subtly reinforce the sponsor’s value proposition, rather than generating neutral insights. Evidence also shows that physicians participating in non-interventional post-marketing studies tend to prescribe the studied drug more frequently: a large German cohort study found a *7%–8% increase in prescriptions during the study* and a *6%–7% increase in the following year* (1, 13, 20). While it is acceptable that physicians adapt prescribing practices during formal trials, poor-quality RWE alone is unlikely to drive this behavior. Nevertheless, the selective framing of registry data to highlight unmet needs can blur the line between education and promotion.
7. *Capability gaps:* In many pharmaceutical companies, RWE functions are underdeveloped at global headquarters or local affiliate level, where resources may be concentrated in medical affairs teams without formal analytical training or spread thin across therapeutic areas. Affiliates and medical affairs teams often lack dedicated epidemiology or biostatistics expertise, relying instead on external vendors or adapting clinical trial–style approaches to observational settings. This can result in studies with weak design, inadequate adjustment for confounders, or superficial analyses that prioritize feasibility over validity. Industry reflections confirm that limited internal capacity remains a barrier to robust non-interventional research (13).

Although many examples arise from registry-based research, comparable weaknesses—such as lack of pre-specification, incomplete confounder adjustment, and selective dissemination—are also observed in EMR- and claims-based non-submission studies. The issue, therefore, extends beyond registries to the broader ecosystem of observational evidence produced outside regulatory oversight.

Comparative analyses illustrate the risks of such practices. For instance, in diabetic kidney disease, comparisons between trial and real-world cohorts highlight mismatched populations; in oncology, restrictive inclusion criteria often reproduce biases seen in trials, reducing generalisability (17). These oncology RWE studies typically aim to contextualize clinical-trial outcomes in routine practice rather than replicate them; however, by retaining restrictive inclusion and exclusion criteria, they often limit generalizability and undercut the promise of real-world research. Similarly, in oncology, expanding eligibility criteria has been shown to improve external validity, yet non-submission real-world oncology studies often fail to mirror such inclusivity, restricting populations in ways that serve feasibility or sponsor convenience rather than scientific rigor (18, 19).

The cumulative effect of these shortcomings is a large body of evidence that looks scientific but lacks the safeguards necessary for credibility. Unlike regulatory-grade RWE, these studies are not subject to systematic bias assessment, independent review, or transparency requirements. This blind spot risks perpetuating low-quality evidence under the guise of scientific legitimacy and erodes trust among clinicians, patients, and policy makers alike.

## Why it matters: implications for science, policy, and patients

The shortcomings of non-submission RWE are not a minor methodological concern; they have tangible consequences across the healthcare ecosystem.

For *clinicians*, the widespread dissemination of non-submission RWE shapes perceptions of treatment effectiveness, safety, and disease burden. When studies are poorly designed or selectively reported, they can reinforce misconceptions, bias prescribing behaviors, or create misplaced confidence in therapeutic strategies. In dermatology and oncology, for example, registry analyses with inadequate adjustment have been cited in promotional contexts as evidence of systemic disease burden or unmet need, subtly influencing treatment adoption without providing robust support (15, 18). Comparable risks arise from EMR- and claims-based studies when endpoints, data completeness, or adjustment methods are inadequately defined, further compounding interpretability challenges. Alongside these risks, many rigorously conducted non-submission RWE studies have provided critical insights—for example, clarifying natural histories of rare diseases, mapping treatment pathways, and generating foundational evidence that informs guideline development.

For *patients*, the implications are equally concerning. Patients often participate indirectly, by contributing data through electronic health records, registries, or claims, with the expectation that their information will advance knowledge and improve care. Regardless of the data source, when that data is used in studies of limited quality or promotional intent, it represents a breach of trust (5, 13). Worse still, weak evidence can slow progress if it crowds out higher-quality analyses, or if misleading conclusions seep into guidelines and shared decision-making tools (14).

In addition to routinely collected data, patients increasingly contribute directly through patient-reported outcomes (PROs), symptom diaries, and digital health tools. The collection of PRO data often requires sustained engagement, time, and emotional investment from participants. Ethical stewardship of such contributions demands not only methodological rigor in instrument selection, timing, and analysis, but also transparency regarding how PRO data will be used and communicated. Failure to collect PROs where clinically meaningful, or collecting them in a methodologically weak or underpowered manner, may undermine both scientific validity and patient trust. Moreover, involving patients in study design and endpoint prioritization—where feasible—can strengthen relevance, interpretability, and legitimacy of real-world evidence.

For *policy makers* and *payers*, the proliferation of low-quality RWE muddies the evidence base used to inform healthcare resource allocation. While HTA bodies and regulators apply increasingly strict filters to submission-grade evidence (3, 4), non-submission RWE often circulates freely in scientific and policy discourse without similar safeguards (13). This creates asymmetry: evidence intended to support reimbursement decisions is tightly regulated, while the much larger body of RWE that frames disease narratives and clinical practice remains unchecked. Such asymmetry not only distorts policy debates but can also diminish the perceived credibility of RWE as a policy instrument when low-quality outputs dominate public and professional communication channels.

At a broader level, these practices *erode trust in the credibility of RWE as a field*. Initiatives such as target trial emulation, transparency templates, and international harmonisation seek to raise standards and demonstrate that observational research can match RCTs in credibility under the right conditions (6, 10, 12). Yet the coexistence of high-quality regulatory RWE with a vast volume of unregulated, non-submission studies risks undermining those efforts. If clinicians and policy makers perceive RWE as inconsistent or biased, the legitimacy of the entire enterprise suffers.

Finally, there is an *ethical dimension*. Generating RWE consumes resources—patients’ data, clinicians’ time, and public trust. Using those resources to produce evidence of questionable validity is not a neutral act; it represents an opportunity cost. Each poorly conceived registry, EMR, or claims analysis displaces the potential for a more rigorous study that could genuinely inform practice and policy. In this light, the unregulated majority of RWE is not merely an academic blind spot, but a barrier to the responsible use of real-world data in healthcare. This responsibility is particularly acute when patients actively contribute PRO data, as weak methodological execution not only wastes resources but diminishes the value of patient engagement itself (21–23).

## Proposed framework for oversight

The framework proposed here applies primarily to industry-sponsored, non-submission RWE—including registry-, EMR-, and claims-based studies used for scientific communication, awareness, or medical education. Nevertheless, its principles—transparency, pre-registration, and accountability—are equally relevant to academic, payer, and other non-industry observational research seeking to maintain credibility and consistency.

Addressing the credibility gap in non-submission RWE does not require replicating the entire machinery of regulatory science. Instead, it calls for pragmatic safeguards that raise the floor of quality without paralysing legitimate scientific communication. Three pillars are proposed:

1. *Protocol Registration*: All non-interventional studies, whether intended for submission or not, should be registered in publicly accessible databases such as the EU PAS Register, ClinicalTrials.gov, or ENCePP. Registration of objectives, endpoints, and analysis plans would create a transparent record that reduces selective reporting and strengthens accountability. Industry statements, such as those from EFPIA, already support this step for hypothesis-evaluating treatment effect (HETE) studies (13). Extending this expectation to all RWE, regardless of regulatory intent, data source, or sponsor type–would represent a modest but impactful reform toward greater transparency.
2. *Analytical Transparency:* Transparency does not require open-sourcing every line of proprietary code, but it does require clarity on methods, assumptions, and definitions as well as explicit attention to data quality, governance, database provenance, and use of a prespecified statistical analysis plan (SAP). Initiatives like STaRT-RWE, HARPER, and REQueST provide structured templates for reporting study design, endpoint definitions, and data provenance (10). Companies should adopt these tools routinely for non-submission studies, ensuring that clinicians, reviewers, and policymakers can assess credibility. Academic and public-sector research groups can also benefit from applying similar transparency templates, promoting shared standards across all RWE. Where possible, code snippets, variable definitions, and sensitivity analyses should be made available, particularly when studies are published in peer-reviewed outlets.
3. *RWE Governance, Accountability, and Capability Building:* Most pharmaceutical companies already operate Global Review Committees (GRCs) or equivalent structures to review clinical trial outputs and major publications. Expanding these committees to review all non-submission RWE, however, risks creating an unmanageable bottleneck. Experience shows that assigning every abstract, poster, or local registry analysis to a central review body leads to delays, reviewer fatigue, and superficial oversight.

A more effective model is to establish *study-specific governance and accountability*. Dedicated study teams—comprising epidemiologists, biostatisticians, medical leads, data managers, and statistical programmers—should be accountable for the statistical analysis plan (SAP), the study report, and all downstream communication materials. Communication materials would then undergo an additional, focused review by scientific communication teams to ensure consistency and quality, without duplicating full methodological review at the committee level.

This model also addresses a broader structural issue: *capability gaps at global and local levels*. Too often, non-submission studies are initiated and “owned” by functions without adequate analytical expertise, leading to weak or inconsistent design. To mitigate this, companies should invest in *capability building and resource hubs*. Locating study teams in countries with strong talent pools and lower operating costs can provide scalable access to skilled epidemiologists, statisticians, and programmers, while maintaining global oversight. This dual approach—study team accountability combined with strengthened analytical capacity—provides assurance that non-submission studies are conducted to a consistent scientific standard, without overwhelming senior committees or relying on *ad hoc* vendor support. These principles can also be adapted by academic or payer organizations conducting non-submission RWE to promote consistent oversight and reproducibility. Building internal epidemiological and biostatistical expertise—whether within pharmaceutical companies, academic institutions, or payer organizations—is not merely an operational improvement but a credibility investment that signals long-term commitment to scientific standards in real-world research.

Furthermore, there is a case for robust monitoring structures—whether industry-wide or academic consortia—to minimize dissemination of low-quality RWE. Educating clinicians and decision-makers on how to critically appraise such evidence is equally important to ensure that only high-quality, actionable findings influence practice.

Implementing this framework would require investment, but it is not without precedent. Registries such as ENCePP already facilitate protocol transparency, while internal medical review boards are a familiar feature of trial governance. Moreover, structured templates and harmonisation efforts are proliferating, providing a ready-made toolkit for industry adoption (12). The challenge is not a lack of methodology, but the absence of expectation. By making these three safeguards routine across all RWE—whether destined for HTA dossiers, congress posters, academic reports, or awareness campaigns—the research community can raise credibility standards without stifling scientific productivity.

Implementation pathways could include coordinated expectations from regulators, HTA agencies, and industry associations. For example, regulatory authorities could extend existing transparency guidance to encompass broader categories of non-interventional research, while HTA bodies could signal preference for evidence originating from registered protocols. Industry consortia and professional societies may also play a role in normalizing registration standards through codes of practice. While legislative reform may not be immediately necessary, stronger alignment of expectations across stakeholders would gradually shift registration from optional to normative practice.

## Conclusion

The ongoing debate about the role of real-world evidence in healthcare has too often focused on regulatory submissions and HTA processes, leaving the much larger corpus of non-submission studies underexamined. These studies—produced for awareness, communication, education, or exploratory purposes—represent the bulk of what clinicians encounter at congresses and in medical affairs materials. Yet their methodological safeguards are minimal, and their oversight is inconsistent at best. This imbalance creates a credibility gap that threatens to undermine the legitimacy of RWE as a whole.

High-quality examples from regulatory pilots, harmonisation initiatives, and innovative methodological programs demonstrate that observational research can achieve rigour comparable to RCTs when supported by governance and transparency. However, without extending even basic safeguards to non-submission studies–whether registry-, EMR-, or claims-based–the scientific community risks perpetuating a two-tier system: rigorous, auditable evidence for regulators, and an unregulated majority for everyone else.

The reforms proposed here—protocol registration, analytical transparency, and governance boards—are not radical innovations but pragmatic adaptations of existing practices. Implemented consistently, they would raise the floor of quality across all RWE, aligning the expectations for non-submission studies with those already recognized for regulatory evidence. More importantly, they would protect patients’ contributions, clinicians’ trust, and the credibility of RWE as a field. The same principles can be meaningfully applied beyond the pharmaceutical industry, ensuring that academic and payer-driven RWE also upholds shared standards of validity and transparency.

Ultimately, the value of RWE lies not only in the data itself but in the integrity of its generation and use. Extending accountability to the unregulated majority is therefore not a matter of compliance, but of professional responsibility. If real-world data are to guide real-world decisions, then all real-world studies—submission or not—must meet a baseline standard of quality and transparency.
